# On representing the prognostic value of continuous gene expression biomarkers with the restricted mean survival curve

**DOI:** 10.18632/oncotarget.6121

**Published:** 2015-10-14

**Authors:** Kevin H. Eng, Emily Schiller, Kayla Morrell

**Affiliations:** ^1^ Department of Biostatistics and Bioinformatics, Roswell Park Cancer Institute, Buffalo, NY, USA

**Keywords:** dichotomization, model diagnostics, prognostic marker, restricted mean survival, survival analysis

## Abstract

**Motivation:**

Researchers developing biomarkers for cancer prognosis from quantitative gene expression data are often faced with an odd methodological discrepancy: while Cox's proportional hazards model, the appropriate and popular technique, produces a continuous and relative risk score, it is hard to cast the estimate in clear clinical terms like median months of survival and percent of patients affected. To produce a familiar Kaplan-Meier plot, researchers commonly make the decision to dichotomize a continuous (often unimodal and symmetric) score. It is well known in the statistical literature that this procedure induces significant bias.

**Results:**

We illustrate the liabilities of common techniques for categorizing a risk score and discuss alternative approaches. We promote the use of the restricted mean survival (RMS) and the corresponding RMS curve that may be thought of as an analog to the best fit line from simple linear regression.

**Conclusions:**

Continuous biomarker workflows should be modified to include the more rigorous statistical techniques and descriptive plots described in this article. All statistics discussed can be computed via standard functions in the Survival package of the R statistical programming language. Example R language code for the RMS curve is presented in the appendix.

## INTRODUCTION

In clinical gene expression studies, one objective of model building and analysis is to clearly summarize the prognostic value of a candidate biomarker. Quantitative biomarkers are usually measured on a continuous scale and a researcher would like to present the relationship between low and high risk sets and survival time outcomes.

We analyzed 195 publications (Supplemental Table 1) that used the ovarian cancer data from The Cancer Genome Atlas (TCGA) [1] listed on nih.cancergenome.gov as of January 20, 2015 and found 39 papers (Table 1) whose focus was ovarian cancer (versus pan-cancer or methodological papers), used the mRNA expression array data and presented a Kaplan-Meier (K-M) survival estimate.

**Table 1:** Summary of literature review of articles citing TCGA ovary data

Papers Listed	195
Informatic/pan-cancer/basic science	129
Focus on clinical ovarian cancer	66
Displayed Kaplan-Meier Plots	39
Stratified on genotypes	6
Stratified by clustering	4
Stratified a continuous score	29
Split on Median	16
Split arbitrarily	7
Split by maximum log-rank	3
Quartiles, tails, insufficient	1 each
information	

We observed that genomic data survival plots are frequently based on a continuous score (29/39, 74%) which must be dichotomized to assign patients to risk sets. The most prevalent method is to simply divide the patients into two evenly sized groups (16/29, 55%). Some articles used arbitrary cut points selected during the analysis with no statistical justification given (10/29, 34%).

Unfortunately, it has been *known that unadjusted stratification leads to over-optimistic inference* [2] and it may be the source of poor reproducibility [3]. In particular, searching out the cutpoint that maximizes differences between survival curves is known to be anti-conservative [4] and simply choosing the median without investigating the functional relationship will lead to poor inference [5]. We point interested readers to accessible editorials on the need for categorization [6], as well as the dangers [2] and underlying assumptions [7].

We expect that many investigators have unintentionally overlooked these important statistical points. It remains relevant as our literature review also uncovered two recent methods articles [8] [9] that promote unadjusted search algorithms as a default option. In one article [8], 118 Wald-type tests significant at *p* < 0.01 were subjected to more robust testing against a bootstrap reference; 44% (52/118) of the Wald tests were not significant, underscoring the point that without further investigation these approaches overstate the confidence of the study conclusions. This is not a failure of p-values and the log-rank or Wald tests, but a failure of the application.

One reason for pursuing dichotomization is the difficulty in presenting an attractive survival curve figure familiar to our clinical collaborators. While Cox's regression model [10] is appropriate for continuous data, the meaning of “a 2.0 hazard ratio (Cox model *p* < 0.05) per one standard deviation of RMA normalized expression” is rarely transmuted into months and years, clinical terms, of survival. Further, as Contal and O'Quigley [4] note, dichotomization loses information if the true relationship between hazard and expression is (log) linear; few studies we reviewed employed the relevant functional relationship diagnostics like martingale residual plots [5].

In this work, we will review the options facing the investigator presented with a continuous marker and a survival response. To accommodate the need to pursue both regression and a survival plot, we propose adapting the restricted mean survival statistic to form a curve analogous to a linear regression's familiar “best-fit line.” We illustrate the use of these plots and their flexibility in a series of worked examples. Throughout, we write for the perspective of a non-expert survival analysis end user who likely knows survival analysis by recipe but may be an expert at more sophisticated algorithms used in cross-validation and marker development.

## RESULTS

The TCGA study of ovarian cancer collected gene expression array data on over 500 tumor samples divided into discovery (n=234) and validation sets (n=269). Using the Affymetrix platform data, we note a total of 12,042 gene-level measurements available for analysis. Each measurement is a continuous feature which is assumed to have a mounded log-normal distribution. The units of expression are arbitrary without a specific curve to register fluorescent intensity to RNA quantity. At issue is whether any one of these gene expression measurements is a good predictor for ovarian cancer progression-free survival (PFS). As our focus is to highlight the analysis of continuous expression markers, we will ignore the issue of known clinical prognostic variables throughout this section. We consider the multiple comparisons problem as ancillary for our analysis, it remains a serious issue in high-dimensional data analysis.

### Concordance/discordance between linear, median-split and non-linear models

A typical first screen is to select genes that have a univariate association with PFS. Of the 12,042 genes, some 1,111 (9%) have significant univariate Cox model score tests (*p* < 0.05). The average significant hazard ratio in the deleterious direction is HR=1.4 (95% of the significant set are between 1.07-2.57) and HR=0.73 (0.34-0.90) in the protective direction. As we summarize in Table 2, the clinical magnitudes are close to 2.5 months (the small size is not unexpected given these are single gene models). To produce a survival curve plot, we might stratify each gene at its observed median creating equally sized high and low risk groups. If we do so, we may be dismayed to discover that for 511 (46%) of these, the Kaplan Meier curves will show no significant difference (log-rank test *p* > 0.05).

**Table 2:** Frequency of significant linear (Cox regression), categorical (median-split Kaplan-Meier and log-rank test) and non-linear models in TCGA data

Method			Months PFS			
Continuous Cox Model	Kaplan-Meier	# of genes	RMS Dynamic Range	D Median split KM	Non-linear Cox Model	Interpretation
ns	ns	10455	0.68	0.85	5%	Not significant.
p<0.05	p<0.05	511	2.66	2.89	6%	Both methods identify differences.
p<0.05	ns	600	2.36	1.38	4%	Effect lost when considering KM analysis.
ns	p<0.05	476	1.47	2.46	14%	Effect is not significant. KM sees a difference. False positive or non-linear.

RMS: Restricted mean survival, PFS: Progression-free survival, KM: Kaplan-Meier, ns: Not significant

We recapitulated this loss of significant effect in simulated data (Supplemental Table 2) assuming n=100 samples, a standard normal covariate, linear effect and exponential failure time; at HR=1.35, the Cox model has 80% power, the median split's log-rank test has power 63% and will miss 25% of the significant continuous models. At HR=1.41, the Cox model has 90% power while the log-rank's 72% power and misses 21% of the significant models.

This apparent discrepancy and loss of power is attributable to the fact that dichotomization at the median has averaged out good signal coming from the Cox regression's log-linear model for the hazard. Conversely, we might consider the set of non-significant Cox model results and apply the median-split method. If we do so, we will find 476 genes (4%) which appear to have been missed by the Cox model. It turns out that these genes have weak linear model signals and their importance may be inflated by the dichotomization.

### Martingale residual diagnostics and the functional form of the model

We investigated this set further and determined that this set does have an unusual number of significant Cox regression models with (polynomial) non-linear effects (tested via smoothing spline as described in the methods): 14% of these cases may be genuine signal versus 5% in the other cases.

**Figure 1:**
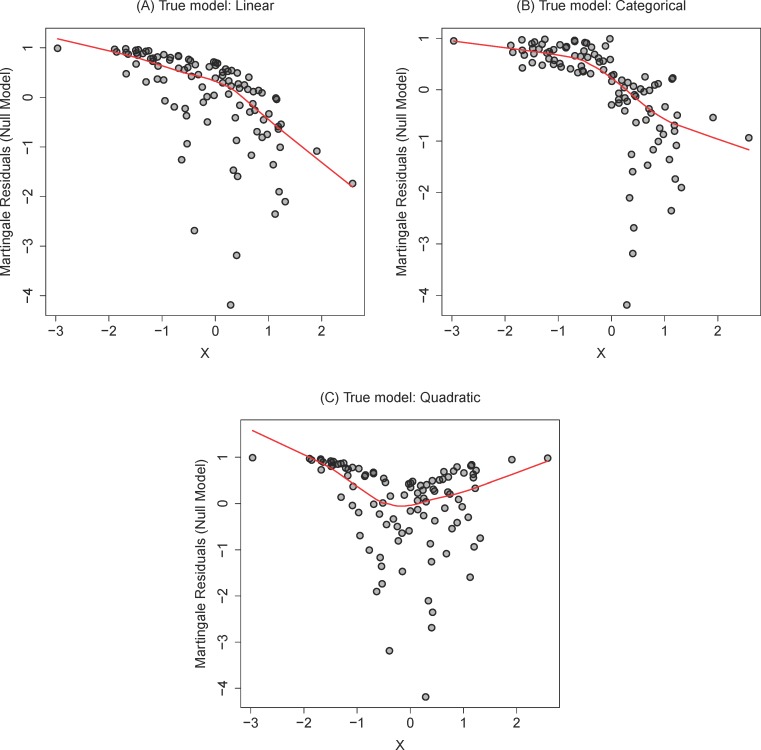
Representative martingale residual diagnostic plots with smoothed mean estimators using data simulated under **A.** linear, **B.** categorical, and **C.** quadratic models. Only the categorical model is appropriate for dichotomization and display via Kaplan-Meier estimate.

In general, a martingale residual diagnostic (described in detail in the methods) can provide empirical evidence for a linear, categorical, or non-linear effect. Using exponentially distributed simulated data, Figure 1 displays three diagnostic plots indicating linear (Figure 1A), categorical (Figure 1B) and non-linear (Figure 1C) regression effects using a “running mean” lowess smoothing estimate. The lowess estimate does not always capture categorical features well; noting that the smoothing line in Figure 1B looks much like the linear function, focusing on the spread of the data points on either side of zero should convince the investigator that a step function (a threshold at zero) is a good model. In practice, if we determine that any model other than the categorical is the best fit, we should avoid using the Kaplan-Meier plot.

### RMS curves summarize Cox model estimated survival curves

**Figure 2:**
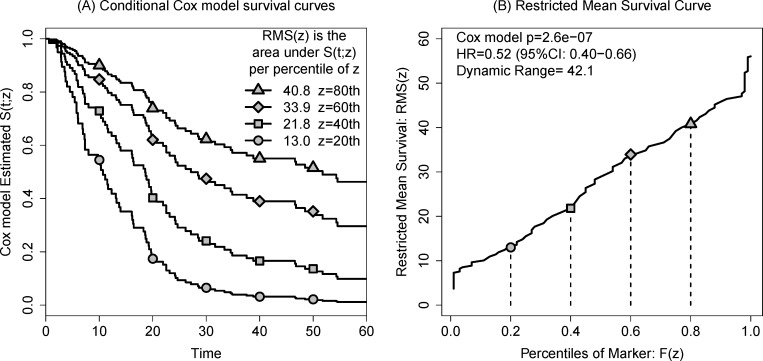
Illustrating the construction of the restricted mean survival (RMS) curve **A.** Conditional Cox model survival estimates $\hat{S_{\text{PH}}}\left(t;z\right)$ at quintiles of Z for simulated data. RMS is the area under each curve (AUC) bounded on the right by τ=60 months. **B.** Plotted as a function of the marker percentile, the RMS curve spans the range of estimable mean survival times for this Cox model. Plotting characters are added to help the reader identify the estimated survival curve and its AUC between panels.

To avoid having to choose between a model that fits well and a model that plots well, we propose that investigators consider a graphic we call the restricted mean survival (RMS) curve that summarizes a fitted Cox model. Consider the following simulated data scenario. We generated exponential failure times under a log linear proportional hazards model and fit a Cox regression model to illustrate the use of the covariate-adjusted survival curve (Figure 2A). In this figure, the survival curve is drawn for four hypothetical patients whose covariate level is assumed to be the 20^th^, 40^th^, 60^th^ and 80^th^ percentiles. As described in the methods section in detail, the area under any one curve is the restricted mean survival time given the corresponding covariate level. If these areas are considered continuously as a function of the covariate values, we produce a second plot (Figure 2B) that we call the RMS curve. We use plotting characters to identify the corresponding points between panels. This curve summarizes the effect of the covariate directly as a function of survival.

While the plot appears non-linear, it should be noted that RMS curves are based on the same log-linear proportional hazards assumptions underlying the Cox regression model: the curve is itself a graphical summary of the model. Thus the model p-values and summary statistics can be displayed with the curve. In the case that a non-linear Cox regression model is considered, the corresponding RMS curve may be highly non-linear.

By converting the continuous model into a dichotomous one, all patients with high scores (or low scores) are given the same estimated survival times. This means that patients with extreme values are treated no differently from those with intermediate values and those patients who happen to fall on either side of the median will have dramatically different prognoses even though they may have very similar scores. We expect these are undesirable properties in a clinical estimate.

### The TCGA193 Marker: hazard ratio and median split

As a concrete case study, we consider the prognostic t-score comprising an individual-specific, two-sample t-test based on the expression of two sets of genes (good and poor) totaling 193 features developed by the TCGA study [1]. We denote this candidate prognostic marker TCGA193 and investigate how we might present its clinical value. We do not intend our remarks to comment on how TCGA193 was developed or initially presented; it is a familiar and readily accessible example. Our analysis is based on the supplementary data from the updated paper Verhaak and colleagues [11].

When scaled to have mean zero and unit standard deviation (sd), the TCGA193 score is associated with overall survival (OS) in the validation set (n=269) via Cox regression. A unit sd change increases the relative hazard by 1.27 (95%CI: 1.06-1.53), p=0.011. The clinical magnitude of this increase is unclear: what does a 27% increase in hazard mean in terms of months of survival?

The median split procedure divides the independent validation (n=269) set into a high set (n=135, median 36.2 months) and a low set (n=134, median 47.5 months); the log-rank test between them is significant (p=0.0372). Given that the median time to recurrence is 18 months and the across-study survival at 60 months is 30.5%, we would gauge a range of 11.3 months to be clinically important.

We might report that the Cox model based on the binary, median-split score provides a hazard ratio estimate of 1.41 (95%CI: 1.02-1.96, model likelihood ratio test p=0.0374) where the p-values differ due to numerical errors, but this is redundant as the point of the median split is to place the marker on a clinical and not relative hazard scale; the consistently significant p-values are judging the same evidence, so presenting both may be misleading.

### Median split is inconsistent with a symmetric, unimodal marker distribution

**Figure 3:**
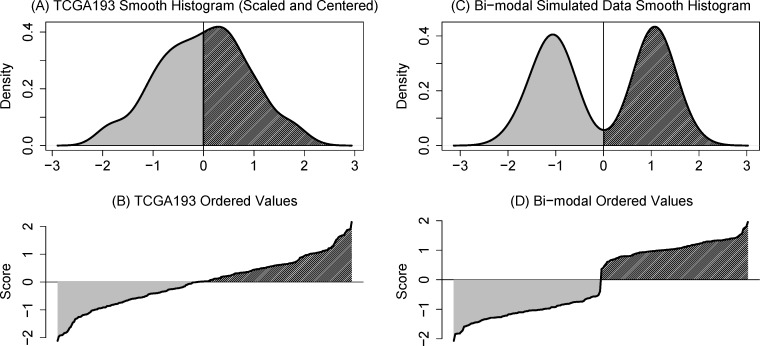
**A.** Discrepancy between Cox model and median split is greatest around the sample mode. **B.** Smoothed density of TCGA193 signature shows a strong mode. **C.** The trace, a plot of ordered values, seems to suggest a cut point. **D.** Smoothed histogram of simulated bimodal data (ideal for a cutpoint). **E.** Corresponding trace plot shows a “cliff” and not a smooth slope.

In Figure 3A, we show the smoothed histogram (density) of the TCGA193 signature which emphasizes that the data are unimodal and mounded around zero. The corresponding “trace plot” (Figure 3B) of the ordered marker values might be shown to imply there is a natural cut point, however consider that the histogram for ideal data for dichotomization should clearly show modes as in Figure 3C. The trace plot corresponding to two modes (Figure 3D) looks nothing like the unimodal plot; we should be looking for sharp jumps in these traces and not a smooth transition across the threshold.

This situation should not be unusual: the extensive literature on normalizing gene expression focuses on producing a reasonably log-normal distribution and the negative binomial distributions assumed to underlie RNAseq data are unimodal. For markers that depend on multiple genes, or procedures like the prognostic-t (i.e., a two-sample t-test), it is likely that a central limit theorem applies, compounding the likelihood that a subgroup-defining threshold will be artificial.

**Figure 4:**
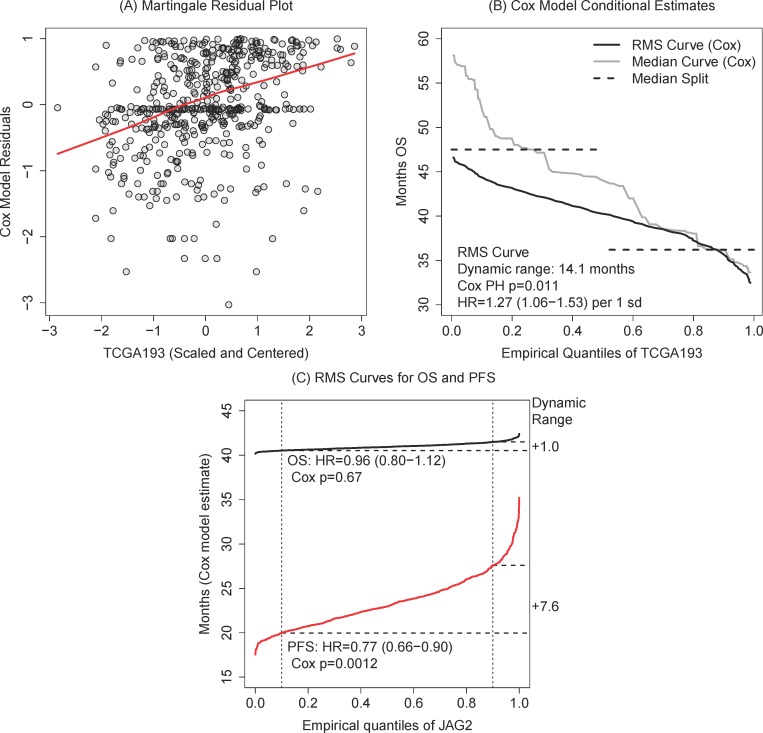
**A.** Diagnostic martingale residual plot with a lowess smoother supports a linear effect for TCGA193. **B.** RMS curve plot expresses the clinical time range covered by Cox model estimate in comparison to the conditional median and median-split estimates. **C.** Over quantiles of JAG2, we plot significant RMS curves for PFS but not OS. This demonstrates a potential way to identify informative genes for ovarian cancer: genes associated exclusively with PFS may be more likely to be treatment-related.

Figure 4A shows the martingale residual plot from a Cox regression model [5] which offers one way to investigate the functional relationship between the marker and the log hazard (an alternative is spline-type modeling, [12]). Plotting the marker values by residuals, we use a lowess smoother to get a non-parametric estimate of the functional form: in this case, a linear effect and not a piecewise constant (assumed by the median split) appears highly appropriate.

### Using the RMS curve

Recalling that we left the continuous, linear Cox model described by a HR=1.27 increase per sd, we use the RMS curve restricting the estimate at 60 months to describe the range of model predictions. This estimate can be computed directly in the R survival package [13] and self-contained, generic R code examples are given in the Supplemental Material.

In Figure 4B, we can read directly that the highest marker values have an expected survival around 32 months versus 47 for the lowest values. This is similar to the median-split (dashed lines), but smooth around the median marker values. We also plot the conditional median (grey line) and find that it agrees with the mean between the 0.60 and 1.00 quantiles of TCGA193 (these are likely the most important patients to identify). The estimates diverge for lower TCGA193 values where the restriction on the RMS curve (at 60 months) is apparent. We interpret this to mean that the conditional median (and the median split) are over-estimates in this range due to particularly long survivors (the maximum observation time is 180 months, the maximum observed death time is 150 months). The RMS curve then provides a clinical check on the estimates by focusing our attention on the first 60 months of survival; plotting both the restricted mean and the (unrestricted) median appears to be good practice.

Given the bell-shaped distribution of TCGA193 (Figure 3A), it is important to note that a woman whose TCGA193 value is −0.22 (48^th^ percentile) and a woman with score +0.02 (52^nd^ percentile) will have a difference in prognosis of more than 10 months. Again it seems undesirable to have such a significant change in prognosis for a small change ( < 5%) in score.

### Multiple outcomes and RMS curves

We note that multiple RMS curves may be plotted together: consider both the OS and progression-free survival (PFS) curves for the same gene. We switch to gene JAG2, a ligand in the Notch signaling pathway highlighted in the original TCGA data paper [1], as a more interesting example. In Figure 4C, JAG2 has a significant association (Cox p=0.0012) with PFS but not with OS (p=0.67). Hazard ratios are scaled to changes in 1sd of expression: for PFS, HR=0.77 (95%CI: 0.66-0.90). The magnitude of the association is captured by the curve and by the summary dynamic range using the 10th and 90th quantiles: a 0.77 hazard ratio translates to about a 7.6 month increase in PFS across 80% of the variation in JAG2.

This pattern of association (with PFS, not with OS) may be a useful way to identify genes associated with treatment: primary ovarian cancer treatment is uniform (maximal debulking followed by platinum/taxane adjuvant chemotherapy) while post-progression treatment varies (interval/secondary debulking, various lines of chemotherapy) in a non-standardized fashion. It is likely that whatever genetic effects associated with OS are strongly confounded by this unmeasured secondary treatment process.

### Multiple RMS curves and treatment prediction

Ovarian cancer patients frequently relapse and undergo multiple lines of chemotherapy. If the initial disease-free interval is longer than 12 months, the choice is a platinum-based regimen. If the interval is shorter than 6 months, the patient is highly unlikely to respond to platinum. In between, the treatment is uncertain. For illustration, we considered patients who relapsed, who were treated with a platinum/taxane regimen (a repeat of the primary therapy) or an alternative topotecan regimen, and who survived to continue a third line of chemotherapy. Response to treatment is measured as the number of months from the completion of the therapeutic regimen until the next event: a further relapse or death. As a marker to select between treatments, we used the apoptosis marker described in our previous work [14].

**Figure 5:**
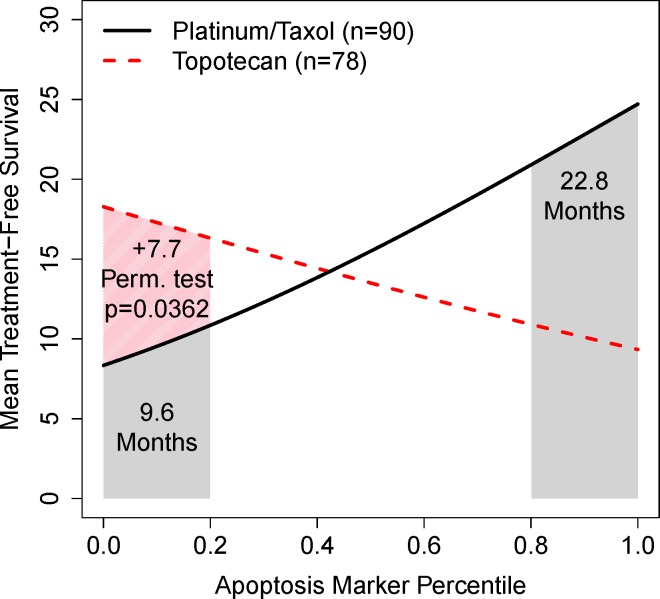
Two example RMS curves for treatment-free intervals under one of two drug regimens after first relapse of advanced ovarian cancer within 6-12 months of adjuvant therapy The shaded regions reflect the expected survival of the top/bottom 20% of patients showing the clinical value of the marker. It is easy to read the added value of the topotecan regimen.

As the apoptosis marker increases, Figure 5 shows that response to platinum/taxane treatment increases (solid line): the lowest 20% of patients have an expected mean of 9.6 months response to therapy while the highest 20% have a 22.8 month response. Because of percentile scale, it is easy to read that the 1st quartile, median, and 3rd quartile marker patients have a mean survival time of 11, 15, 20 months respectively.

The topotecan marker does the reverse; noting that the platinum/taxane treatment is the default, we might propose that patients with the lowest 20% apoptosis marker level might try topotecan instead: the expected benefit is an extra 7.7 months. We might assign significance to this result by reporting the p-value from adding the interaction term in the Cox regression model (likelihood ratio p=0.00768), but a test of the 20% strategy can be made directly by permuting the drug class labels (platinum/taxane versus topotecan). A gain of at least 7.7 months was seen in 392 of 10,000 permuted samples implying that it is unusual and of statistical interest.

Plotting multiple treatments on the same graph was proposed by Janes and colleagues [15] with respect to candidate biomarkers and response rates. Following their logic, we might read the intersection point (around 40%) to be the strategy threshold between the topotecan and platinum/taxol treatments.

## DISCUSSION

Prognostic biomarkers are an important part of precision medicine and their translation relies on flexible and interpretable descriptive techniques. We have discussed a way to report both a Cox regression model and have an attractive plot that accurately summarizes the model. This RMS curve can be calculated from standard software and can be adapted to fit the clinical question under study. While the admonitions against dichotomization have a long history, we believe it is important to continue to point out that careless dichotomization is a serious problem and that there exist viable and possibly better alternatives.

We summarize the recommendations from our literature review and illustrations:

- Report the Cox model hazard ratio for the marker re-scaled to unit standard deviation so that effect sizes can be compared across studies.
- It should be noted that Kaplan-Meier curves make minimal assumptions on the form of the survival curve, but including “Cox p-values” or hazard ratios implicitly attaches a proportional hazards interpretation to the curves.
- Conversely, avoid presenting the conditional survival function estimated under proportional hazards without careful reasoning about which covariate levels to represent. If the curves are used, clearly label them as model-based because they will appear misleadingly ideal. Avoid plotting the censoring marks for these curves. A trained eye should detect “parallelism” due to the same support of unique failure times in each curve.
- Consider the martingale residual plot to develop support for or against a linear model or categorization.
- When the true model is linear, dichotomization-based estimates will be unstable around the cutpoint. This error is especially acute when the cutpoint is close to the empirical mode.
- Avoid selecting cut points arbitrarily because it is unclear how to account for the error in selection. Cross-validation [16] may help find an unbiased cutpoint, but it will not solve the significance problem.
- If a test statistic or p-value search algorithm is used, the reported p-value should be adjusted [4].

A problem with conditional RMS and the median estimates derived from Cox's regression model is a lack of test procedures; this should not detract from the adoption of a descriptive and practical point estimate and it should not support the use of bad estimators.

While we focus on regression-derived markers or prognostic scores, a careful reader will extrapolate that many of our observations extend to scores based on linear functionals of gene expression space. Our development is strongly tied to the analysis of gene expression data and our conclusions may not generalize to every application of survival analysis.

## MATERIALS AND METHODS

### Notation and assumptions

We begin with a typical survival analysis regression situation: suppose we observe a random sample

{(Y_i_,δ_i_,x_i_)}_(i=1)^n

where Yi is a complete survival time when δi=1 and a right censored time otherwise and xi is a p-vector of covariates (i.e., gene expression values, clinical features) which may be continuous or categorical. When convenient, we will make reference to the unique ordered failure times t(1)< t(2) < … < t(D) and the set of patients at risk at time

t_j_: R(t_j_) = {i: t_i_≤t_j_}.

We assume that the investigator has some procedure (say, ζ(.)) that converts xi into a scalar score comprising an operationalized biomarker, z_i=ζ(x_i ) a real number. We review approaches to dichotomization and key parts of the proportional hazards (PH) model before introducing a new tool: the restricted mean survival curve.

### Dichotomization procedures

When the observed set of z_i_ is dichotomized into two mutually exclusive sets *L* = {*i*:*z_i_* ≤ *μ*}and *H* = {*i*:*z_i_*> *μ*}, these sets can be treated like independent samples and separate K-M curves can be estimated in the usual fashion. Further, the significance of differences between the curves might be quantified by the log-rank statistic.

It has been noted that this procedure may be invalid if it does not account for error in the choice of cut point 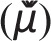. The following are candidate procedures for selecting $\overset{\vee }{\mu }$ and reporting its significance:

**Median-Split.** Choose $\overset{\vee }{\mu }$ to be the sample median. The test procedure is the simple log-rank test. If only the median is considered, the log-rank is a valid test; but if the true model is not dichotomous at the median the choice introduces serious bias.
**Maximum Split.** Consider all possible cutpoints C_k_ and let S_k_ be the associated log-rank statistic. Referenced earlier, various papers show this estimate is anti-conservative and leads to overconfident inference. $\overset{\vee }{\mu }$ is the C_k_ corresponding to the maximum |*S_k_*|. The p-value is based on the corresponding log-rank test; this is known to be anti-conservative.
**Contal and O'Quigley.** Choose $\overset{\vee }{\mu }$ as in the maximum split procedure. A test statistic that accounts for error in the choice of split point is
  $$Q=\frac{\underset{k}{\text{max}}|S_{k}|}{s\sqrt{D-1}}$$
  $$s^{2}=\frac{1}{D-1}\sum_{i=1}^{D}\left[1-\sum_{j=1}^{i}\frac{1}{D-j+1}\right]$$
  where D is the number of distinct death times [5]. Under the null hypothesis of no effect, Contal and O'Quigley [4] showed that Q has a limiting distribution related to a Brownian Bridge; the p-value is approximately P(Q > q)≈2exp(−2q^2^) when q > 1. For practical reference, values of Q larger than 1.358 or 1.949 are significant at *p* < 0.05 and *p* < 0.001 respectively. The exact formula is given in both references.

### Proportional hazards regression

For the casual survival analysis reader, we define S(t; z) to be the bi-variate survival function that depends on time and score. Cox's proportional hazards (PH) regression model [10] assumes that the hazard function can be factored into a time and covariate component:

$$\text{log}h(t;z)=\text{log}h_{0}(t)+\beta z.$$

Using standard software this univariate PH model depends only on the estimate $\hat{\beta }$ that maximizes a partial likelihood (the reader is referred to textbooks, e.g., [5]). It follows that for some z_0_, the hazard ratio h(t;z_0_+1) / h(t;z_0_) = exp(β), is the relative change in hazard for a one unit increase in Z. The units of Z should be reported because re-scaling Z will affect $\hat{\beta }$. For gene expression, this scale is likely un-interpretable and it may be good practice to normalize Z to have unit standard deviation. The result would be read as the hazard ratio for a one standard deviation change in expression.

### Conditional Cox model-based survival curves

Under the previous PH model, it follows that the survival function is assumed to have the form:

$$S_{\text{PH}}(t;z)={\left[S_{0}(t)\right]}^{\text{exp}(\beta z)}.$$

An estimate of S_PH_(t; z) depends on both $\hat{\beta }$, computed via partial likelihood, and an estimate of S_0_(t). One estimate of the baseline survival function is based on Breslow's estimator [17] which again depends only on $\hat{\beta }$:

$$S_{B}(t)=\text{exp}\left\{-\sum_{t_{j}\leq t}\frac{1}{\sum_{i\in R(t_{j})}\text{exp}(z_{i}\hat{\beta })}\right\}$$

forming a non-increasing step function. Link [18] described a linearly-interpolated estimate as well as its confidence intervals. The conditional survival curve is sometimes called the covariate-adjusted survival curve.

This formula means it is possible to compute a survival curve for each value of the continuous score. The natural problem is that we may choose any value of z that we wish including values outside the range of observation. These curves may be misleading [19] for an audience that expects K-M curves: by construction, for various values of z, $\hat{S_{PH}}\left(t;z\right)$ will show “parallelism” (e.g., the curves in Figure 1A will never cross and will decrease at the same times for all curves) for distinct values of z and they cannot show the assumption-free behavior of K-M curves.

### Restricted mean survival

The area under a given survival curve S_PH_(t; z) reflects the mean (model-based) survival time for a subject with marker value z. The restricted mean survival (RMS) time depending on z is

$${\text{RMS}}_{\tau }(z)=\int_{0}^{\tau }S_{\text{PH}}(t;z)\text{dt}$$

where the restriction time τ > 0 is required to account for an excess of survivors. The selection of τ is discussed in general in other work [20]. The RMS is interpretable as the expected life experienced out of τ units of time and therefore reflects the value of the model in plain language clinical units of time.

### Restricted mean survival curve

To show the impact of continuous biomarkers on response rates, Janes and colleagues [15] developed a graphical plot that lent itself to comparisons of multiple groups for illustrating treatment strategies. Analogously, we focus on RMS(z) as z varies to produce a graphable curve:

RMS(.)={(Fz),RMS(z)):−∞<z<∞,0≤F(z)≤1}

where F(z) is the cumulative distribution function (CDF). This curve will be monotone increasing when β < 0 and monotone decreasing when β > 0. The case that β=0 corresponds to a horizontal line. Because we have re-scaled the x-axis to percentiles (using F(z)), marker-defined subgroups can be identified by selecting a range and the area under the RMS curve on this domain is proportional to the expected survival of this subgroup.

An estimator of RMS(z) using *τ=max_i_ Υ_i_ δ_i_*is:

$$\hat{\text{RMS}}(z)=\sum_{i=1}^{D}\left(t_{\left(i+1\right)}-t_{\left(i\right)}\right)S_{B}{\left(t_{\left(i\right)}\right)}^{\text{exp}(\hat{\beta }z)}$$

where t_(i)_, i=1,…,D are the unique ordered failure times. This leads to an estimator of RMS(.) using the empirical CDF, $F_{n}(z)=n^{-1}\sum_{i=1}^{n}I\left\{z_{i}\leq z\text{ and }\hat{\text{RMS}}\text{(z)}\right\}$. The difference between the maximum and minimum RMS values, the dynamic range,

$$\text{DR}=|\hat{\text{RMS}}(\text{min}z)-\hat{\text{RMS}}(\text{max}z)|$$

may be a useful statistic that estimates the total amount of survival at issue with respect to this marker. The minimum and maximum might be replaced by appropriate quantiles to avoid misleading tail behavior.

### Cox model conditional median plot

Because the estimated conditional survival function $\hat{S_{\text{PH}}}\left(t;z\right)$ can be computed for any z, we might also estimate the conditional median for each value of z. The use of multiple quantiles and curves has been discussed for data exploration [19], and we might consider adapting the methods above for a median-based curve:

$$\left\{\left(F(z),t_{m}(z)\right):0<F(z)<1,\hat{S_{PH}}(t_{m};z)=1/2\right\}$$.

Because this estimate does not depend on τ, it provides a way to gauge the influence of the restriction. Further, the standard statistical intuition about means and medians applies as survival times tend to be right-skewed.

## SUPPLEMENTARY TABLES






