# Supplementary material for: On representing the prognostic value of continuous gene expression biomarkers with the restricted mean survival curve
Source: Oncotarget. 2015 Oct 14;6(34):36308–18. doi: 10.18632/oncotarget.6121 (PMC4742179; doi:10.18632/oncotarget.6121)
Supplement: Supplementary file 3 [file oncotarget-06-36308-s003.pdf]

Supplement: Example RMS curves in R


```
## Example of RMS Curve Calculation
## Using R package Survival
require(survival)
data(ovarian)

# Parameters for RMS
tau      <-60  # Restriction point
n.grid   <-100 # For plotting purposes

# Set up data using age as the marker
OS       <-Surv(ovarian$futime/30.5,
                ovarian$fustat)
marker   <-ovarian$age
marker.pp<-seq(from=0,to=1,length=n.grid)
marker.qq<-quantile(marker,marker.pp)
fitdat.df<-data.frame(marker=marker)
newdat.df<-data.frame(marker=marker.qq)

# Calculations
cox.model<-coxph(OS~marker,data=fitdat.df)
rms.calc <-summary(survfit(cox.model,
              newdata=newdat.df),rmean=tau)
rms.mean <-rms.calc$table[,"*rmean"]

# RMS Curve
plot(marker.pp,rms.mean,type="l",
     xlab="Marker Percentile")

# Model summary
summary(cox.model)

## Because age is directly interpretable,
plot(marker.qq,rms.mean,type="l",xlab="Age")
```
